# High frequency of complex *CFTR* alleles associated with c.1521_1523delCTT (F508del) in Russian cystic fibrosis patients

**DOI:** 10.1186/s12864-022-08466-z

**Published:** 2022-04-01

**Authors:** Nika V. Petrova, Nataliya Y. Kashirskaya, Tatyana A. Vasilyeva, Natalia V. Balinova, Andrey V. Marakhonov, Elena I. Kondratyeva, Elena K. Zhekaite, Anna Y. Voronkova, Sergey I. Kutsev, Rena A. Zinchenko

**Affiliations:** 1 Research Centre for Medical Genetics, Moscow, Russian Federation; 2 Laboratory of Genetic Epidemiology, Research Centre for Medical Genetics, Moskvorechie St., 1, 115522 Moscow, Russian Federation; 3 N.A. Semashko National Research Institute of Public Health, Moscow, Russian Federation

**Keywords:** CFTR, *Cis*-mutation, Complex alleles, Cystic fibrosis, Russian patients

## Abstract

Cystic fibrosis (CF, MIM# 219,700) is an autosomal recessive disease caused by pathogenic variants within the *CFTR* gene. It was shown that genetic variants located *in cis* can affect disease severity or treatment response because of additive or epistatic effects. Studies on the prevalence of complex alleles in Russian CF patients have just begun.

**Aim**

To evaluate frequencies and genetic background of complex alleles carrying c.1521_1523delCTT (F508del) and c.1399C>T (L467F), c.2562T>G (T854=) or c.4389G>A (Q1463=) *in cis*; to determine clinical consequences of complex allele c.[1399C>T;1521_1523delCTT] ([L467;F508del]) in Russian CF patients.

**Methods**

Sequencing of coding regions of *CFTR* gene and analysis of polymorphic markers in CF patients carrying F508del variant. Comparing of clinical features in two groups patients having genotypes [L467F;F508del];[F508del] (group 1) and [F508del];[F508del] (group 2).

**Results**

Frequency of [L467F;F508del] allele linked to 2–2–21–6–17–13 haplotype was 4.42%, of [F508del;T854=;Q1463=] allele linked to haplotype 1–2–21–6–17–13 – 2.2% in F508del chromosomes. No differences in disease severity in patients carrying complex allele [L467F;F508del] and patients homozygous for F508del was found.

**Conclusion**

The frequency of complex alleles associated with F508del was at least 6.6% in Russian CF patients, which should be taken into account for the decision on optimal treatment options with CFTR modulators.

Cystic fibrosis (CF) is a common hereditary autosomal recessive disease in the Caucasian population, the molecular cause of which are mutations in the *CFTR* gene. More than 2,100 pathogenic, probably pathogenic, benign, and of unclear clinical significance sequence variants of the *CFTR* gene are registered in Cystic Fibrosis Mutation Database (CFTR1) [1]. The CFTR2 database contains a smaller set of variants with proven clinical significance: 360 causing CF, 48 having varied clinical consequences associated with *CFTR*-related disorders or CF; 23 — benign (not causing CF) and 11 with unknown significance [2].

Currently, drugs targeting specific pathogenic variants are being developed for CF personalized treatment. So, it is necessary to clarify the clinical consequences of not yet characterized variants and even well-known variants, for example, c.1521_1523delCTT (p.Phe508del, F508del) (ClinVar accession VCV000007105.51), forming complex alleles. Genetic variants localized *in cis* with known mutation forming the complex alleles can affect both the phenotypic manifestations of the main variant and cause resistance to treatment with targeted drugs due to additive or epistatic effects [3–5]. The spectrum and frequency of complex alleles in different populations vary and have not been adequately studied.

Our study is aimed to determine the frequency and genetic background (haplotypes) of a number of *CFTR* complex alleles carrying the F508del variant in Russian CF patients.

## Methods

Sanger sequencing of the coding regions, intron–exon junctions, 5′- and 3′-UTRs of the *CFTR* gene in DNA samples of 122 unrelated CF patients who are compound heterozygous for F508del and another variant, as well as the sequencing of the exon 11 of the *CFTR* gene in DNA samples of 120 patients homozygous for F508del variant was performed according to the protocol described previously [6]. Sanger sequencing of exons 15 and 27 of the *CFTR* gene in DNA samples of 91 unrelated CF patients homozygous for the F508del variant was performed. All biological samples are deposited into the Moscow Branch of the Biobank “All-Russian Collection of Biological Samples of Hereditary Diseases”.

The study of alleles of polymorphic markers XV2c and KM19 was performed by restriction fragment length polymorphism (RFLP) analysis. Analysis of alleles of polymorphic markers IVS1CA, IVS6aGATT, IVS8CA, and IVS17bCA in the *CFTR* gene was performed by the amplified fragment length polymorphism (AFLP) method. The combination of alleles linked to *CFTR* mutations is further called a haplotype. Haplotype analysis was performed in DNA samples of 91 patients homozygous for the F508del and was carried out according to the previously described protocol [7, 8]. All identified variants were designated according to the reference transcript variant NM_000492.4 of the *CFTR* gene on initial mention followed by legacy nomenclature which is widely accepted in CF research.

The frequency of identified allele was calculated according to the formula ${p}_{i}={n}_{i}/n$ where ${n}_{i}$ is the amount of $i$-th allele, $n$ is the sample size (the number of tested chromosomes). The Wald method was used for calculating 95% confidence intervals (95% CI).

To describe the clinical characteristics of CF patients, data from the 2019 Russian CF Patient Registry (RCFPR) were used [9]. Data from 11 patients with genotype [L467F;F508del];[F508del] (group 1) were available. The group of patients with genotype [F508del];[F508del] (group 2) included 73 patients and was matched for age and sex to group 1.

The RCFPR project was approved by the Ethics Committee of the Research Centre for Medical Genetics on December 20, 2012 (Chairman of the Ethics Committee – Prof. L.F. Kurilo), and patients with cystic fibrosis and/or their representatives signed informed consent.

The following parameters were considered: patient age, age at diagnosis, sweat test parameters, body mass index (BMI; kg/m^2^), spirometric parameters: forced expiratory volume in 1 s (FEV_1_) and forced vital capacity (FVC) of the lungs, presence of microorganism colonization in the bronchopulmonary system (*Pseudomonas aeruginosa, Staphylococcus aureus, MRSA, Burkholderia cepacia* complex*, Achromobacter* spp.*, Stenotrophomonas maltophilia*, non-tuberculosis mycobacteria, gram-negative microflora), pancreatic insufficiency, complications (meconium ileus, CF-related diabetes, osteoporosis, allergic bronchopulmonary aspergillosis (ABPA) and so on) and treatment.

Statistical analysis was performed using the STATISTICA v.8.0 program. To compare categorical variables, the Fisher test was used; for quantitative variables, the Mann–Whitney *U* test was used. The significance level of *p*-value ≤ 0.05 was considered significant.

## Results

### Complex allele [L467F;F508del]

Analysis of *CFTR* sequence in 122 patients compound heterozygous for F508del variant and another pathogenic variant, c.1399C>T (p.Leu467Phe, L467F) (ClinVar accession VCV000053246.12) variant was detected in 5 cases in a heterozygous state. Analysis of patients’ parents showed that L467F and F508del variants were *in cis*, forming complex allele. To clarify the proportion of [L467F;F508del] complex allele out of chromosomes carrying F508del variant, 120 patients homozygous for F508del variant were analyzed. [L467F;F508del] complex allele was detected in a heterozygous state in 11 patients. Thus, the proportion of the [L467F;F508del] complex allele out of chromosomes carrying F508del variant appears to be 4.42% (95% CI 0.0230–0.0654). The analysis of haplotypes linked to [L467F;F508del] complex allele revealed 2–2–21–6–17–13 haplotype.

The summary table with the observed allele frequencies by the group during the study is presented in Table 1.

**Table 1:** Distribution of F508del complex alleles containing L467F variant

Genotype	No. patients	No. F508del alleles	F508del allele structure	No. (%)
F508del/other	122	122	L467F;F508del	5
			F508del	117
F508del/F508del	120	240	F508del;L467F	11
			F508del	229
	overall	362	L467F;F508del	16 (4.42%)
			F508del	346 (95.58%)

### *Complex allele [F508del;T854*=*;Q1463*=*]*

Two complex alleles c.[1521_1523delCTT;2562T>G] ([F508del;T854=]) and c.[1521_1523delCTT;4389G>A] ([F508del;Q1463=]) are described in the literature [4]. Sequencing analysis of DNA of patients compound heterozygous for F508del for these described variants of the *CFTR* gene showed that these complex alleles can also occur in Russian CF patients. Variant T854= (ClinVar accession VCV000043577.17) was found 32 times in the heterozygous state in a sample of 97 patients (proportion 16.67%; 32/194), and variant Q1463= (Clinvar accession VCV000093157.18) was found 29 times in the heterozygous state in 102 patients (its proportion was 14.22%; 29/204). Segregation analysis in families of these patients was not possible due to the lack of parental DNA. Therefore, the search for T854= and Q1463= variants was performed in 91 CF patients, homozygous for F508del. Two patients were identified as heterozygous for either T854= or Q1463= variants, and one was homozygous for both variants. Familial analysis of heterozygous patients confirmed that T854= and Q1463= variants are located on the same allele, forming a complex allele [F508del;T854=;Q1463=]. Thus, the proportion of this complex allele in chromosomes carrying F508del was 2.20% (4/182; 95% CI 0.0007–0.0433). It is linked to a single haplotype 1–2–21–6–17–13 of polymorphic loci as well as to variants c.2619+85_2619+86delAT and c.*125T [8] in exon 15 and 3′-UTR, correspondently.

The summary table with the polymorphisms/mutations identified, in how many patients, by the group during the study is presented in Table 2.

**Table 2:** Distribution of F508del complex alleles containing T854= and Q1463= variants

Genotype	No. patients	No. F508del alleles	F508del allele structure	No. (%)
F508del/F508del	91	182	F508del;T854=;Q1463=	4 (2.2%)
			F508del	178 (97.8%)

### Study of clinical features of complex allele [L467F;F508del]

The results of the study of the patients’ health status in 2019 are presented in Table 3.

**Table 3:** Clinical and demographic characteristics of CF patients

Characteristics	**Group 1**	**Group 2**	***P***-value
	**p.[L467F;F508del];[F508del], *n* = 11**	**p.[F508del];[F508del], *n* = 73**	
**Age at follow-up, years**			
M ± SD	15.0 ± 9.3	15.5 ± 10.7	*p* = 0.963
Me (IQR); Me (Q25;Q75)	12.6 (15.2); 12.6 (9.3; 19.7)	12.0 (18.1); 12..0 (6.7; 24.6)	
**Sex distribution**			
Males	5 (45.5%)	34 (46.6%)	*p* = 0.945
Females	6 (54.5%)	39 (53.4%)	
**Age of diagnosis, years**			
M ± SD	3.0 ± 6.4	2.0 ± 4.6	*p* = 0.564
Me (IQR); Me (Q25;Q75)	0.7 (1.9); 0.7 (0.2; 2.0)	0.4 (1.2); 0.4 (0.2; 1.3)	
**Sweat test**			
Sweat conductivity	*n* = 6	*n* = 37	*p* = 0.875
	112.5 (26.0); 112.5 (105.0; 123.0)	117.0 (25.0); 117.0 (101.0; 124.0)	
**Diagnosis**			
Proportion of patients diagnosed by clinical features, %	5 (45.5%)	36 (50.7%)	*p* = 0.782
Proportion of patients diagnosed by neonatal screening (IRT test-positive), %	6 (54.5%)	33 (46.5%)	
Proportion of patients with false negative neonatal screening, %	0 (0.0%)	2 (2.8%)	
**Meconium ileus**			
no, %	11 (100.0%)	65 (91.5%)	*p* = 0.370
Surgical treatment, n (%)	0 (0.0%)	6 (8.5%)	
**Microbiology**			
Chronic *Pseudomonas aeruginosa*, n (%)	2 (18.2%)	35 (48.6%)	*p* = 0.059
Intermittent *Pseudomonas aeruginosa*, n (%)	2 (18.2%)	7 (10.0%)	*p* = 0.422
* Staphylococcus aureus*, n (%)	8 (72.7%)	49 (68.1%)	*p* = 0.756
* Burkholderia cepacia complex*, n (%)	1 (9.1%)	5 (6.9%)	*p* = 0.798
* Nontuberculous Mycobacteria****,*** n (%)	0 (0.0%)	1 (2.8%)	*p* = 0.683
* Stenotrophomonasmaltophilia*, n (%)	1 (9.1%)	9 (12.5%)	*p* = 0.746
* Nontuberculous Mycobacteria*, n (%)	0 (0.0%)	13 (18.3%)	*p *= 0.122
* Achromobacter spp.*, n (%)	1 (9.1%)	7 (9.9%)	*p* = 0.936
* MRSA*, n (%)	0 (0.0%)	4 (5.6%)	*p* = 0.420
**Lung function**			
FEV_1_, % of predicted	*n* = 6	*n* = 38	*p* = 0.584
	63.7 ± 16.8	70.2 ± 30.1	
FVC, % of predicted	*n* = 6	*n* = 38	*p* = 0.081
	70.3 ± 14.1	84.3 ± 23.9	
**Complications**			
ABPA, n (%)	0 (0.0%)	0 (0.0%)	-
DIOS, n (%)	0 (0.0%)	1 (1.4%)	*p* = 0.694
Fluid and electrolyte disorders, n (%)	0 (0.0%)	3 (4.2%)	*p* = 0.490
CF-related diabetes (treated with insulin), n (%)	0 (0.0%)	4 (5.6%)	*p* = 0.423
Pneumothorax, n (%)	0 (0.0%)	2 (2.8%)	*p* = 0.574
Hemoptysis, n (%)	1 (9.1%)	1 (1.4%)	*p* = 0.121
OccurMalignancy, n (%)	0 (0.0%)	0 (0.0%)	-
Osteoporosis, n (%)	1 (12.5%)	12 (22.6%)	*p* = 0.514
Polyposis of the paranasal sinuses, n (%)	5 (45.5%)	24 (34.3%)	*p* = 0.473
Amyloidosis, n (%)	0 (0.0%)	0 (0.0%)	-
**Liver disease**			
cirrhosis with portal hypertension, n (%)	1 (33.3%)	3 (21.4%)	*p* = 0.381
cirrhosis without portal hypertension, n (%)	1 (33.3%)	0 (7.1%)	
liver disease without cirrhosis, n (%)	1 (33.3%)	10 (71.4%)	
**Fecal elastase 1**			
< 200 µg/g one time, n (%)	5 (100.0%)	36 (97.3%)	*p* = 0.300
≥ 200 µg/g one time, n (%)	0 (0.0%)	1 (2.7%)	
**Mortality**			
Number of deaths, n (%)	0 (0.0%)	5 (6.8%)	*p* = 0.374
Age at death, years	-	28.9 (26.4; 31.4)	

As can be seen, the groups did not differ in the studied clinical characteristics. It should be noted that there were cases of false-negative neonatal screening (IRT test) in group 2. Meconium ileus was not found in group 1. Chronic *Pseudomonas aeruginosa* was more common in group 2 (*p* = 0.059). Lung function in group 2 (in terms of FVC, %) exceeded that of group 1 (*p* = 0.081), despite the higher frequency of chronic *Pseudomonas aeruginosa*. In addition, 5 cases of death during the follow-up period were registered in group 2 (Table 3). Causes of death: 1 person – complications after lung transplantation, 4 patients—lung disease. But these differences did not reach the level of significance.

## Discussion

The F508del variant is the most frequent pathogenic variant of the *CFTR* gene, the frequency of which is 62.73% in Europe (ECFS Patient Registry Annual Data Report 2018) [10] and 53.14% in Russia (RCFPR 2019) [9]. The development and use of CFTR modulators are primarily aimed at treating a large group of patients carrying this variant. The RCFPR 2019 includes data on 909 patients, homozygous (30%), and 1400 patients (46.2%), compound heterozygous of the F508del variant. The presence of complex alleles can alter residual CFTR protein activity, leading to resistance to targeted therapies. The frequency and spectrum of such variants in many populations remain to be investigated.

An example of this fact is the study of Baatallah N., et al. that described 21 complex alleles (with one or two variants *in cis*) with F508del mutation [4, 5]. Some of these variants are very rare [3], while others make up a significant proportion of F508del alleles in a number of populations: [F508del;I1027T] is more than 5% in Southern Brittany [11]; [A238V;F508del] – 6% in Southern Italy [12].

The effect of some *cis*-variants on CFTR protein biogenesis, response to targeted therapy, and disease severity of F508del homozygous patients has been proven [13, 14]. For example, enhanced exon skipping was observed with I203and D924N which would reduce the amount of residual CFTR available in cells for correction [5]. Additionally, L467F was found to reduce CFTR maturation by half and, when associated with F508del, prevented the response to corrector treatments and led to resistance to Lumacaftor rescue [4]. The [F87L;I1027T;F508del] complex allele abolished the Lumacaftor corrector effect [15].

The effect of the A238V variant on the clinical picture of the disease in F508del homozygotes was shown [12, 13]. CF patients carrying the complex allele [A238V;F508del] had a more severe systemic inflammatory process due to a lung dysfunction, compared to patients who carried only the F508del variant. It was also noted that the A238V variant leads to patients’ resistance to targeted therapy.

Thus, the presence of complex alleles increases the complexity of assessing residual CFTR protein function, CF patient phenotype, and response to targeted therapy. It was shown that L467F was found to reduce maturation efficacy and the [L467F;F508del] CFTR did not respond to the corrector/potentiator treatment. L467F and F508del are both located within the NBD1 domain of CFTR and both lead to folding defects hindering protein maturation [4].

In Russia, special systematic studies devoted to the analysis of complex alleles of the *CFTR* gene are limited, identified randomly in sporadic cases. Only M. Ivanov, et al. identified four [L467F;F508del] complex alleles in the heterozygous state in 184 adult Russian CF patients [14]. The proportion of this complex allele in F508del chromosomes was 6% (4/66).

In our study the proportion of [L467F;F508del] complex allele was 4.42% that wasn’t significantly differed from M. Ivanov’ data [16] (*z* = 0.5808. The value of *p* = 0.56192, *z*-score test for two proportions). Allele [L467F;F508del] was linked to a single haplotype of polymorphic loci. It should be noted that allele 2 of XV2c polymorphism is rare on chromosomes carrying F508del variant. In tested F508del homozygous samples, allele 2 of XV2c polymorphism was found on 16 of the 182 chromosomes (i.e., in 8.8%), of which 11 carrying [L467F;F508del] complex allele (*z* = 8.4686, *p* < 0.00001). This pointed to the common origin of the complex allele in the Russian population. To assess the response to CFTR modulator therapy, a systematic and detailed study of the phenotype of patients carrying this complex allele, including the dynamics, is necessary.

As well as in Ivanov’s study [14], no significant differences in disease severity were found between patients carrying complex allele and patients homozygous for F508del. However, in our study mortality was higher in the group with genotype [F508del];[F508del] than in group [L467F;F508del];[F508del]. An increase of the sample may reveal differences between the studied groups.

Baatallah N. et al. [4] and B. Chevalier, A. Hinzpeter, 2020 [5] described two different complex alleles [F508del;T854=] and [F508del;Q1463=]. It was shown that at least synonymous variant T854= could alter protein function, a rare codon could alter protein synthesize rates hence proper protein folding and function [17]. In our study we found both variants T854= and Q1463= *in cis* with F508del forming a single complex allele [F508del;T854=;Q1463=] with a relatively high proportion.

## Conclusion

Thus, for the first time in Russia, the frequency of complex alleles carrying functionally significant variants potentially affecting resistance to the response of patients to targeted therapy has been evaluated. In Russian CF patients, the proportion of the [L467F;F508del] and [F508del;T854=;Q1463=] complex alleles from all chromosomes carrying the F508del variant was 6.6%. A systematic detailed study of the phenotype of patients over time with analysis of response to targeted therapy, as well as a more in-depth molecular genetic confirmatory diagnosis of CF patients in each case, is required.

## Data Availability

The datasets used and/or analyzed during this study are available from the corresponding author on reasonable request.
